# Monkeypox: Treatment, Vaccination, and Prevention

**DOI:** 10.7759/cureus.33434

**Published:** 2023-01-06

**Authors:** Sehrish Shah, Darshna Fulmali

**Affiliations:** 1 Anatomy, Jawaharlal Nehru Medical College, Datta Meghe Institute of Medical Sciences, Wardha, IND

**Keywords:** management, epidemiology, eczema vaccine, self- limiting, monkeypox

## Abstract

In regions where the disease is endemic, Monkeypox (MPV) transmission related to healthcare has been seen on numerous occasions. This disease has episodes of occurrence in certain regions around the globe, such as in the Democratic Republic of Congo’s (DRC) Tshuapa region. Here, the disease was found with a prevalence of 0.35 per 1000, as per data collected by the Centers for Disease Control and Prevention (CDC) of the United States (US). Data also shows approximately 100 confirmed cases of MPV for every infection among Healthcare Workers (HCWs). These findings and scientific research on burns, superficial wounds, herpes, eczema vaccine, and other conditions indicate that MPV sufferers might get an advantage from medical care to lessen the effects of weakened skin and mucosa. This should involve guarding delicate anatomical areas like the eyes and genitalia, maintaining enough hydration and nourishment, and preventing and treating consequences like secondary bacterial diseases. In the DRC, this disease was first recognized in 1970. Since then, it has spread to numerous nations around the globe and gained substantial epidemiological significance. The most recent epidemic has taken place in 2022 worldwide. The viruses that cause MPV and cowpox are currently regarded as emerging. Because of the rise in international travel, the popularity of exotic pets, and the decline in smallpox vaccination rates, they pose a significant danger of spreading. Although it is believed that this viral illness will eventually go away on its own, the possibility of the pandemic raises several serious problems for the general public's health. In addition to providing a broad overview of the Monkeypox Virus (MPXV), this study will detail the epidemiology, clinical hallmarks, assessment, and treatment of MPV sufferers.

## Introduction and background

The camelpox, cowpox, vaccinia, and variola illnesses are all caused by members of the genus *Orthopoxvirus*, which also contains the Monkeypox Virus (MPXV). The World Health Organization (WHO) has determined that this virus is the most common *Orthopoxvirus* impacting human populations since smallpox has been eradicated [1]. In the Democratic Republic of the Congo (DRC), where there are thought to be over 1000 cases of Human Monkeypox (MPX) annually since 1958, healthcare personnel is frequently exposed to the illness [2]. A member of the *Poxviridae* family is Monkeypox (MPV). Additionally, the *Poxviridae* family is separated into the *Entomopoxvirinae* and *Chordopoxvirinae* subfamilies. In 1958, an epidemic of MPXV in monkeys in a Danish lab led to its initial discovery. In 1968, a nine-month-old boy contracted MPV just two years before smallpox was eradicated in DRC [3]. Further, it was recognized as a disease affecting people. Although MPXV is spread in the Congo Basin to a more significant extent, the occurrence of MPV in human beings, flora and fauna has also been found in other Central and West African nations. But, because these native regions have insufficient infrastructure and resources necessary for endemic and ecological investigations, surveillance is challenging [3].

Although it only causes minor infections, Molluscum contagiosum is more prevalent in youngsters than in adults, and there is a possibility of Sexually Transmitted Infections (STIs) or, in cases of excess, Acquired Immunodeficiency Syndrome (AIDS) [4]. In the spring of 2003, this virus made its initial appearance in the countries of the United States (US) and caused a large number of illnesses in the Midwest of the US. This drew the virus to more notice [5]. We now have a better grasp of the epidemiology of MPX because of the construction of reliable surveillance technologies, which also made it possible to monitor risk groups passively. Healthcare Workers (HCWs) were subsequently identified as a group at increased risk for infection. Examining whether medicinal countermeasures created for smallpox may be successfully employed for MPX is made possible by the platform of scrutiny for MPX in Tshuapa [2].

After African mammals were transferred from Ghana to the US, where they infected native American prairie dogs and disseminated the infection to five states, there was the first MPXV epidemic outside of Africa, with a total of 47 recorded human cases [3]. The general lack of vaccination in the 1980s has increased the human population's vulnerability to MPXV infection. As a result, there are worries that the MPXV could be employed in bioterrorism. Limiting contact with people and animals who are infected and respiratory exposure to these people are essential components of effective prevention [5]. Besides collecting data on efficacy and well-being, field administration of the vaccine offers additional logistical knowledge that can be used in MPX and smallpox preparedness planning. It is important to note that the MPXV is the only *Orthopoxvirus* to have caused an epidemic in the US since smallpox was eradicated. It is wise to be ready for smallpox and MPX outbreaks [2]. The present state of knowledge regarding MPV will be reviewed in this article, with a focus on epidemiologic traits, clinical traits, diagnosis, and therapy [5].

## Review

Methods

**Figure 1:**
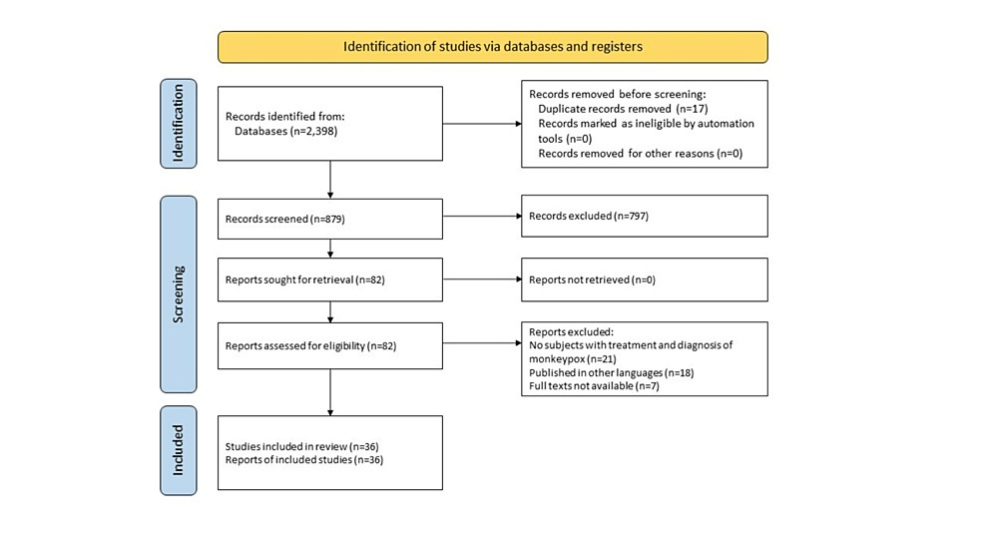
Prisma Flowchart

We undertook a systematic search through PubMed in August 2022 using keywords such as “monkeypox”, “human monkeypox”, “diagnosis of monkeypox,” and “treatment of monkeypox”. We additionally searched for key references from bibliographies of the relevant studies. Reviewer independently monitored the retrieved studies against the inclusion criteria in the beginning, based on the title and abstract and then on full texts. We included studies that assessed the diagnosis, treatment, prevention, and vaccination of monkeypox. For inclusion, both published and unpublished studies in the English language were considered. We excluded studies that were published in other languages because of resource limitations or if the full-text articles were unavailable to reviewers. We finally included 36 articles in this review article after the exclusion of the articles as shown in (Figure 1).

Epidemiology

In Nigeria, a four-year-old child was the first to contract Monkeypox Virus (MPXV), and her mother became infected with the virus two years later. Residents of Ihie Umduru, which is in the current state of Abia, were affected, and people assumed that the mother contracted the disease from her child. In a similar vein, a 35-year-old man from Omifunfun in Nigeria became infected with MPXV for the third time in 1978. Between 1971 and 1978, out of 10 cases reported, three cases were confirmed, and no deaths occurred [6]. In 1981, serotherapies were carried out in Sierra Leone, Cote d'Ivoire, Congo, and Zaire on the people who were not vaccinated. In a sampling on the DRC, 19.2% of individuals tested positive for the *Orthopoxvirus* by the generic serologic test known as Hemagglutination Inhibition Testing (HAI). One hundred seventy-eight HAI-positive samples were examined with the Radioimmunoassay Adsorption (RIAA) test, a more accurate Human-Monkeypox (MPX) serologic test. Of the 178 sera tested, 27 were positive for Monkeypox (MPV) [7]. In five states of the Midwestern United States (US), 71 cases of MPV were documented between May and July 2003 [8]. However, in September 2017, a contagious skin rash condition that resembled MPV was discovered in an 11-year-old child from the country's southern region, and it was later found that this was due to the MPV virus [9].

More than 80% of MPX cases in Africa involve youngsters under the age of 10. 90% of those afflicted live in villages with fewer than 1000 inhabitants, making it primarily a disease of rural populations [10]. The Nigeria Centre for Disease Control (NCDC) informed WHO of a few cases as of October 27, 2017, and patients in Abuja, Enugu, Bayelsa, and Akwa Ibom have since been confirmed [6]. On May 6, 2022, a British national who had recently returned from Nigeria, where the illness is endemic, showed symptoms of the illness, making this the first case of MPXV in the United Kingdom (UK) [11]. Beginning in May 2022, there has been the largest-ever outbreak of MPV in non-endemic nations. Given the rate of transmission to non-endemic countries, understanding MPV epidemiology is urgently needed to help therapists, preventive medicine experts, and educationalists to be prepared for any eventuality, even though no cases of MPV have been reported from India until mid-June 2022 [12]. A few minor outbreaks and lone cases of MPX were discovered in the UK (in 2018 and 2019), Israel (in 2018), Singapore (in 2019), and the US (in 2021). All of these cases were connected to travel to Nigeria, where MPX has returned, and the country has reported more than 500 suspected cases since 2017 [13].

Diagnosis

**Figure 2:**
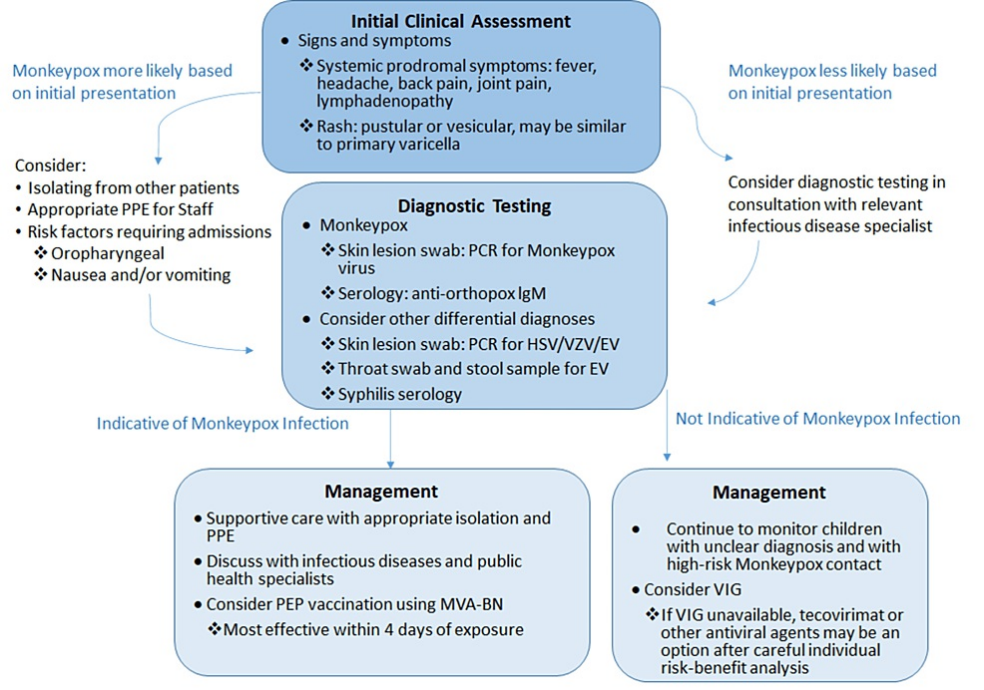
Key characteristics for diagnosing and treating monkeypox disease Source [17] PPE: Personal Protective Equipment, PCR: Polymerase Chain Reaction, IgM: Immunoglobulin M, HSV: Herpes Simplex Virus, VZV: Varicella-Zoster Virus, EV: Enterovirus, PEP: Post-Exposure Prophylaxis, MVA-BN: Modified Vaccinia Ankara-Bavarian Nordic, VIG: Vaccinia Immune Globulin.

Swabs of vesicular lesions, exudate, or crusts maintained in cool and dry sterile tubes make the ideal clinical samples for laboratory testing. An oropharyngeal or nasopharyngeal swab should be used to obtain a viral culture [14]. Smallpox and other deadly human illnesses have been successfully eradicated thanks to vaccines. We must be alert to these risks because these may create immunological niches for novel developing infections like MPV. Veterinarians, zoologists, and wildlife biologists are also responsible for the early detection, management, and prevention of re-emerging viral zoonotic. These individuals are in addition to clinicians and public health professionals [15]. Laboratory studies, clinical symptoms, and medical history all contribute to diagnosing MPXV infection. The diagnosis of MPV infection includes immunohistochemistry, western blotting, Polymerase Chain Reaction (PCR), and Enzyme-Linked Immunosorbent Assay (ELISA). To exclude other infectious diseases like smallpox, a confirming diagnosis is required. A swab is used to collect sweat from a lesion to capture viral nucleic acids for diagnosis. The MPV genome-specific Real-Time Reverse Transcription-Polymerase Chain Reaction (RT-PCR) assay is then performed using viral Deoxyribonucleic Acid (DNA). According to the WHO, RT-PCR is the go-to test for identifying MPXV during a critical infection [16]. Another possibility is a serological examination. Four to 56 days after rash onset, MPV patients often show detectable levels of Immunoglobulin M (IgM) anti-orthopox antibodies or a fourfold increase in Immunoglobulin G (IgG) antibody titers between critical and improved specimens. Based on any report of subjection to MPV in the past 21 days, cases may be categorized as suspicious, likely, or proven (Figure 2). Typically, the results of histological analysis of skin lesions are useless since they are non-specific [17].

Treatment

Most individuals recover without treatment since the symptoms of the MPV infection are typically minor. According to the Centers for Disease Control and Prevention (CDC) recommendations, infections with the MPXV do not yet have a specific therapy. However, antiviral drugs that have a smallpox license can also be utilized for treating MPV [16]. In-vitro and preclinical investigations have shown that the antiviral drug Cidofovir (Vistide) is effective against poxviruses by inhibiting viral DNA polymerase [16]. An animal model was used in a clinical trial for the Food and Drug Administration (FDA) approved drug 4-trifluoromethylphenol derivative Tecovirimat (ST-246 or TPOXX®®), which was developed for this purpose. By preventing the intracellular virus' release from the cell, the medication has demonstrated its efficacy in treating infected animals. The Tecovirimat human clinical trial, according to the CDC report, showed that the drug was safe, but there is inadequate information on how well it treats MPXV cases in humans [6].

Since smallpox and MPXV are closely related, the smallpox vaccination also confers cross-immunity to diseases caused by other *Orthopoxviruses*, such as MPV. According to studies, the smallpox vaccine can help prevent MPV by at least 85%. However, nations have gradually stopped giving the smallpox vaccine since WHO declared smallpox extinct in 1980. As a result, those under 40 are no longer protected against MPV by the prior smallpox immunization regimen [18]. Supportive and symptomatic therapy has been used frequently in treating MPXV since then [19]. Since there is little information on the effectiveness of licensed medications against MPXV in people, its usage is likely to be restricted to managing severe cases [20]. Although MPV typically resolves on its own, infants, young children, and those with underlying immune weaknesses may experience more severe sickness or even pass away [21].

Transcriptome analysis has been used extensively in in-vitro studies on the molecular pathogenesis of MPXV infection [22]. For most of those enzymes' substrates, the effects are not anticipated to be clinically significant based on the number of interactions and the length of Tecovirimat administration. Tecovirimat may raise the levels of repaglinide in diabetic patients receiving repaglinide therapy, which could cause hypoglycemia. When given to patients who are taking midazolam, Tecovirimat may cause midazolam levels to drop, necessitating dose adjustments, monitoring of the midazolam impact, or using other sedatives [23]. The FDA has given the smallpox treatment drug Tecovirimat approval because it blocks the primary envelope protein of the* Orthopoxvirus*. It has been discovered to shield nonhuman primate models from fatal infection with the MPXV [24]. The use of the drugs in a few cases of MPX has shown that Tecovirimat is effective while Brincidofovir is useless. *Orthopoxvirus* can acquire drug resistance in cell culture when passaged with either ST-246 or Cidofovir by causing mutations in the F13L or E9L (DNA polymerase) subunits [25].

Vaccination

The first vaccinations were developed using pustules from bovine smallpox before it was even known that the disease was a virus. After then, attenuated strains of smallpox were substituted for the vaccine virus to immunize large populations before smallpox was eradicated [26]. Although the smallpox vaccine looks to be successful, a vaccine specific to MPX is now being developed [27]. Research demonstrates that smallpox immunization effectively shields against MPV and other *Orthopoxvirus* infections [3]. A close relative of the Variola virus is the MPXV. Because MPXV and smallpox antigens are identical, the smallpox vaccine is effective against MPXV. But more than 70% of people alive today have never received a smallpox vaccination [20]. Unchecked replication of the virus in the Human Immunodeficiency Virus (HIV)-positive, immunocompromised population, the lack of smallpox vaccination, the virus' wide host range, undetected circulation in wildlife across all geographical regions, the emergence of virus strains with better adaptations are some of the likely causes of a great extent of the disease [28].

As the number of people receiving smallpox vaccinations declined, we used a constant rate of immunity decrease at the individual level of 1.29% per year [29]. More than 7800 people have received vaccinations in several clinical trials using the Modified Vaccinia Ankara-Bavarian Nordic (MVA-BN) backbone, revealing a safety profile similar to that of other licensed, contemporary vaccines. MVA-BN received approval as a smallpox vaccine in 2013 for use in Europe and Canada and in 2019 for use in the US as a smallpox and MPV vaccine [30]. A "ring vaccination" strategy is employed to immunize persons who have contact with known cases to stop the spread of *Orthopoxvirus*. ACAM2000 vaccination provides immunity to *Orthopoxvirus* infections, such as smallpox and MPV [24]. The American CDC decided on May 24, 2022, to release some of its JYNNEOS vaccines, a live vaccinia vaccine initially authorized for the smallpox virus in 2019. This vaccination is intended for people at increased risk of encountering MPXV [16].

Additionally, the highly attenuated smallpox vaccine LC16m8 with an enhanced safety profile has been created and granted Japanese licensing [31]. For instance, there is a chance of significant side effects, such as eczema vaccinatum, in locations where MPV is an endemic disease due to the unknown prevalence of HIV infection or other types of immunosuppression [32]. Furthermore, many cases of replication-competent Vaccinia Virus (VACV) strains spreading to close contact with vaccine recipients have been documented [33].

Prevention

The battle to control the spread of MPX was handled on various fronts, as described further. Limiting human exposure to suspicious host species must be the priority on the ecological front since data suggests that interhuman transmission spread can't support the persistence of an endemic without frequent zoonotic invasions. This can be done by decreasing human reliance on hosts, especially rodents, as a source of protein and increasing dependence on vegetarian substitutes [34]. To prevent the eviction of reservoir animals, urban growth into recovered forest lands must be carefully considered. Ecological prevention is crucial because future strains may not require repeated introductions if their human transmission potential were to develop [29]. Protecting at-risk populations, such as healthcare professionals, those who know MPXV patients, and people who work in rural regions, would be another front. The CDC suggests that smallpox vaccines be used to obtain this protection [14]. This is the recommended course of action for a patient with a fever and a widespread vesicular or pustular rash [35]. About six cases make up the longest chain of affected individuals, according to data, and compared to smallpox, MPV is less infectious among humans [1].

Immunocompromised people should not receive the smallpox vaccine. Modified Vaccinia Ankara (MVA) vaccines are still being developed, but complete licensure of antiviral therapy options to support preventative efforts should be taken into consideration [36]. Human-to-human infection transmission can be prevented by following infection control techniques such as gloves, protective clothes, surgical masks, and trained staff. In the case of developed nations, if a patient comes to the hospital with MPV symptoms, s/he is to be kept in an isolation room with negative air pressure, if available, or in a private room. Preventions should be taken for droplets, contacts, and general hazards. Personnel in charge of infection control should be called right away. Likewise, in affluent nations, raising knowledge of the illness and its endemic regions among medical professionals is a crucial preventative measure [14].

## Conclusions

In the woodlands of central and western Africa, MPV is most prevalent. The illness is a typical zoonosis, unlike smallpox, which means that the majority of cases are caused by close contact with an animal that is infected. A timely and precise laboratory diagnosis is crucial for managing an outbreak because the symptoms in people can be strikingly similar to those of smallpox, chickenpox, or other kinds of vesiculopustular rash. Due to the resemblance between smallpox and African MPV cases and the population's declining immunity after the termination of smallpox vaccination campaigns, the prospect that MPXV could be used as a biological weapon has been raised. The CDC has designated MPXV as a "select agent," a term for bacteria, viruses, toxins, rickettsia, and fungi. Previously believed to be geographically restricted and unimportant to the United States, this incident has brought attention to the issues with the trade in exotic pets and increased concerns about the growing movement of wild animals around the world as well as other potential carriers of infectious diseases. Other creatures, including prairie dogs, were prohibited from being imported, captured, transported, sold, traded, distributed, and released into the wild. In the May 2022 outbreak of MPV, many countries with confirmed cases had no travel links with the areas endemic to this disease. To ensure public health readiness and create defenses against potential threats, it will be helpful to have a better understanding of the factors influencing the transmission of this virus. Most cases occur in rural Africa, a region with weak health infrastructure and few resources, so it is essential to consider the possibility of underreporting.
